# The Only Natural Remedy

**Published:** 1895-09

**Authors:** 


### THE ONLY RATIONAL REMEDY.

The repeated occurrence of rape in this State, the great increase in this particular crime, and the cropping out here and there of other forms of sexual crimes and misdemeanors, despite the fact that the former is a capital offense, and that a number of criminals have been executed for it—some by hanging, some by mob violence, warns us that the law is inadequate for its prevention, and emphasizes the necessity for a change. Statistics of the last census show that the crime of rape is alarmingly on the increase; and without the statistics we are made aware of the fact by the daily papers, which every day record one or more cases, some plain rape, others with the most shocking accessories; and it is alarming. The law seems to have no terrors for this class; and if ever anything were satisfactorily demonstrated, it is that hanging is a back number, and has failed totally of the end for which it was ever instituted.

Hanging is to be condemned from every standpoint. It not only does not restrain the criminally inclined, but there is reason to believe that it really acts as a suggestion and incentive to others to commit similar crimes.

There is a growing sentiment in favor of abolishing the death penalty for rape and substituting castration. The medical profession are almost a unit in favor of it, and the majority of thinking men with whom we have conversed approve of the proposed change. It is the only rational remedy; and seeing the total inefficiency of the means so long resorted to, we certainly are justified in trying it. The only objections we have ever heard raised against castration for crime is that it is "unconstitutional;" that the Constitution prohibits any cruel and unusual punishment, or maiming of any person.

What is wanted is not *punishment* for the crime, but prevention of repetition, and of hereditary transmission of the tendency; for "punishment" carries with it the associated idea of revenge, and the law has no right to take vengeance; but the object of all law should be, in addition to the protection of society, reform and reclamation.

The reason why capital punishment is ineffective in dealing with this class is, they are diseased persons; they are insane; they are moral monsters, and in all probability, (meaning those cases that violate the dictates of nature, and are the more revolting), are not responsible, but act in obedience to an impulse which they can not control, emanating from a diseased brain center for which they are not responsible, but are indebted to some progenitor—perhaps away back yonder.

To hang, or otherwise put to death a morally insane criminal—if he can be called a criminal—is as irrational as it is useless; it is revolting to every sense of humanity. These creatures are more to be pitied than blamed; and are fitter subjects for treatment than for execution. It is a stain upon the fair page of 19th century civilization, and the day will come when we will blush to own it, as we do now to remember that our ancestors of only a few generations ago burned insane creatures for "witches."

As pointed out in a former article* castration is particularly

* Castration of Sexual Perverts, by F. E. Daniel, M. D., Medico-Legal Journal, Dec., '93.

adapted to this form of crime; in fact, it recommends itself as a rational treatment for all sexual crimes. It meets all the requirements; it prevents a repetition or attempt at repetition of the offense; prevents hereditary transmission of the tendency or predisposition, to other generations, and is thus not only a protection to society now, but insures the future against it. In addition to this, there is strong reason to believe that the operation of removing the glands will exert a curative influence upon the mind, and that it will have the effect of deterring others—as suggested in the paper referred to, on the principle of the singed rat at large amongst his kind.

Thus the operation will be *preventive* and *curative*, and if the element of punishment is to be considered—this would seem to be sufficient punishment to satisfy the most exacting stickler for it. Moreover, the operation performed under anæsthetic influence is painless, and is not attended with danger. The physical system is not affected, or if it be, it is made stronger, and the party is not rendered incapable of doing manual labor.

Therefore, if the constitution prohibits it, *change the constitution.* Amend our laws so as to substitute this humane act, this rational 19th century remedy for crime committed by morally diseased persons, for the inhuman, irrational, barbarous, and worse than useless hanging,—a relic of the dark ages; and let us put a stop to this great and increasing crime, this constant menace to every home and fireside.

The foregoing has been suggested, partly, by reading in the daily press recently the conviction and sentence to death of a white man at Georgetown, Texas—by name Jay Owens—for rape and incest,—the victim being his own child, thirteen years of age. Who does not pity this creature! By a perversion of a god-given sense, through perhaps no fault of his,—but due, in all probability, to a contamination imparted by a debauched ancestor,—he is changed from a man, a proud father, into a monster too horrible to think about. Why hang him? Why not castrate him and put him in the insane asylum, or at work in the State's prison? If capable of reasoning and reflecting,—if not entirely bereft of the moral sense—all those years which he would spend separated from the world as some beast dangerous to the flock, and at work for the benefit of the society he has outraged and whose rights he has forfeited—he would be a prey to his own conscience, the victim of remorse, which would be "punishment," if any have the heart to desire punishment for such pitiful creatures,—a thousand times worse than death!

Certain it is,—society needs to be protected from this source of danger, and has a right to demand it of the State. Hanging is inadequate; does not afford it, and the exigency of the case demands a change; it is the right of every tax payer to demand the enactment of laws that will protect his family as well as his property. Arouse, wake up, ye dreaming theorists and scruplers over forms;—demand of your legislators a law which fills the requirements of the hour, at once rational and effective. As Dr. Blaine said at Denver—in advocating this measure—"Doctors, *keep the ball in motion!*"

